# Neural regeneration in the human central nervous system—from understanding the underlying mechanisms to developing treatments. Where do we stand today?

**DOI:** 10.3389/fneur.2024.1398089

**Published:** 2024-05-09

**Authors:** Christopher Elnan Kvistad, Torbjørn Kråkenes, Sonia Gavasso, Lars Bø

**Affiliations:** ^1^Neuro-SysMed, Department of Neurology, Haukeland University Hospital, Bergen, Norway; ^2^Department of Clinical Medicine, University of Bergen, Bergen, Norway

**Keywords:** central nervous injury, ischemic stroke, multiple sclerosis, regeneration, spinal cord injury

## Abstract

Mature neurons in the human central nervous system (CNS) fail to regenerate after injuries. This is a common denominator across different aetiologies, including multiple sclerosis, spinal cord injury and ischemic stroke. The lack of regeneration leads to permanent functional deficits with a substantial impact on patient quality of life, representing a significant socioeconomic burden worldwide. Great efforts have been made to decipher the responsible mechanisms and we now know that potent intra- and extracellular barriers prevent axonal repair. This knowledge has resulted in numerous clinical trials, aiming to promote neuroregeneration through different approaches. Here, we summarize the current understanding of the causes to the poor regeneration within the human CNS. We also review the results of the treatment attempts that have been translated into clinical trials so far.

## Introduction

Neurological disorders represent a major cause of morbidity worldwide, and the prevalence is increasing (1). Injuries to the human central nervous system (CNS) are irreversible because neurons have severely limited abilities to regenerate. Resident neural stem cells in the periventricular zone of the cerebrum are also largely incapable of replacing dead or degenerated neurons (2). Consequently, acquired CNS injuries often result in permanent disability. This is a common denominator, regardless of aetiology. The lack of therapeutic options for promoting repair are opposed to the enormous burden that CNS injuries cause, both from an individual and socioeconomic perspective.

In contrast to the mature CNS, where axonal regeneration following injury is inhibited, neurons in the peripheral nervous system (PNS) are capable of re-entering the growth program, thereby enabling the axons to at least partly regenerate (3). The general cause for this divergence within the same nervous system is related to the need for preventing ectopic axon growth and aberrant synapse formation in the CNS. While uncontrolled growth after injuries may be detrimental within the brain and spinal cord, this does not cause the same magnitude of complications in single nerve structures, although neuropathic pain is a common complication following PNS injury. In contrast to humans and mammals in general, the CNS of other species, such as fish and amphibians, are capable of regeneration. The poor reparative capacity of our CNS may thus be regarded as an evolutionary trade-off for its high complexity.

An obvious question arising is what specific mechanisms account for the lacking regenerative properties in the mature human CNS, and whether and how these may be reversed. During the last decades, extensive research has identified both intrinsic and extrinsic factors responsible for the limited regeneration within the CNS. Different treatment modalities, including various types of stem cell transplantation, have been tested in clinical studies with the aim of promoting repair. Any therapeutic with the ability to achieve this would likely have a substantial impact on the quality of life for patients with different types of neural injuries. In order to design effective regenerative therapies, there is a need for detailed knowledge concerning the mechanisms preventing this to occur naturally within the CNS.

In this narrative review, we summarize the current understanding of the underlying mechanisms to the poor regenerative potential in the human CNS. We also discuss what specific intra- and extracellular elements are required to achieve meaningful neural regeneration. Finally, we summarize the trial results of the most promising neuroregenerative treatment modalities that have been translated into clinical studies thus far. As CNS injury is multifactorial, we aim to base the review on three different, yet common causes of irreversible CNS injury: spinal cord injury (SCI), multiple sclerosis (MS) and ischemic stroke (IS).

In the following section, we briefly summarize the pathophysiological mechanisms leading to neural injuries in these conditions.

## Pathophysiology of CNS injury in spinal cord injury, multiple sclerosis, and ischemic stroke

### Spinal cord injury

Traffic accidents and falls are the most common causes for SCI, and the incidence is highest among young and elderly individuals (4). The cervical spine is the most frequent site of injury (5). In the acute phase of traumatic SCI, the mechanical blow causes axonal injury, while the disrupted microvasculature and injured blood–brain barrier (BBB) expose the spinal cord to macrophages, neutrophils and lymphocytes and other blood components (6). The ensuing inflammatory response and injury to the BBB leads to oedema, which results in further compression of CNS tissue and necrosis. Following the initial insult during the subacute phase, inflammation, excitotoxicity and impaired vascular autoregulation contribute to additional loss of neurons and oligodendrocytes in numbers that may exceed those caused by the impact of the initial event (7). Reactive astrocytes migrate to the margins of the lesion, where they proliferate, express cell adhesion molecules and differentiate into scar-forming astrocytes. These elongated cells adhere to each other and secrete extracellular matrix molecules to form a glial scar (8). In the following weeks and months during the intermediate and chronic phase, myelin products and cell debris are phagocytized by microglia and macrophages, resulting in the formation of cystic cavitations filled with extracellular fluid and thin bands of connective tissue (7). The mature glial scar surrounding the site of injury forms a barrier that prevents spreading of the inflammation, but also inhibits axonal regeneration.

### Multiple sclerosis

Multiple sclerosis (MS) is an immune-mediated disease of the CNS characterized by inflammation causing multifocal demyelination and subsequent neuronal degeneration. MS is the most common non-traumatic cause of disability in young adults. Globally, some 2.8 million people are affected and the incidence has increased over the past decades (9). MS has been considered a disease triggered by T cell-mediated autoimmune events with peripheral activated immune cells invading the BBB and thereby causing inflammation with secondary axonal degeneration. The beneficial effects of anti-CD20 therapies indicate a central role of B cells in the pathogenic cascade. Recent data show that infection with the Epstein–Barr virus is an important cause due to epitope mimicry, as all MS patients have been infected prior to the disease (10). The diagnostic hallmark of MS are demyelinated lesions in the white and gray matter with loss of oligodendrocytes (11). In the early stages of MS, the lesions are infiltrated with T and B lymphocytes. Initially, axons are largely preserved within the demyelinated lesions where there is some degree of spontaneous remyelination (12). Remyelination occurs after the phagocytosis of myelin products by microglia and infiltrating macrophages. Despite remyelination, the newly formed myelin sheets are thin and leave the axons vulnerable to excitotoxicity and insufficient metabolic support, leading to progressive neurodegeneration and atrophy (13). Chronic lesions that continue to slowly expand, named “smouldering lesions,” contain an expanding ring of activated microglia surrounding the inactive demyelinated area (14). Chronic meningeal inflammation causing subpial damage also contributes to neurodegeneration, especially in progressive MS (15).

### Ischemic stroke

IS is a leading cause of death and a major contributor to disability worldwide. The incidence of stroke in younger people has increased in recent years (16). IS is typically mediated by an arterial blood clot interrupting the blood supply to an area in the brain, thereby causing ischemia. The following ATP depletion leads to an unbalanced intracellular influx of ions and water, culminating in the breakdown of neural cell membranes (17). Free radicals are generated, further damaging cellular content and DNA. Immediate cell necrosis occurs within the most hypoxic areas, which attracts microglia and leukocytes via upregulation of endothelial adhesion molecules (18). The following inflammatory reaction and injury to the BBB cause oedema, which may result in further deterioration of the blood supply, causing further ischemia. Areas of less hypoxic tissue surrounds the necrotic core. The neurons within this “penumbra” may survive for a longer period due to a minimal supply of oxygen and nutrients. However, if blood flow is not timely reestablished, also this area will turn into infarction. In contrast to the necrotic core, these neurons of the penumbra may be able to undergo apoptosis instead of necrosis, minimizing damage and disruption to neighbouring cells. Numbers of activated astrocytes and microglia increase in the peri-infarcted region in the first five days after injury, where they start forming the glial scar, which compartmentalizes the injury site from the remaining healthy parenchyma (19).

Despite marked differences in pathophysiology, SCI, MS, and IS all typically lead to irreversible neural injury, for which there is no current treatment for repair. In the following section we will focus on the mechanisms responsible for the poor abilities of the CNS to regenerate.

## Mechanisms inhibiting regeneration in mature CNS

### Extrinsic inhibition of regeneration

#### A historic perspective

The irreversibility of injuries to the mature CNS has been known for a long time. Already 3,500 years ago, Egyptian physicians accurately described human para- and tetraplegia and the serious prognosis associated with such conditions (20). This was shown at the cellular level at the beginning of the 20th century as Santiago Ramon y Cajal, a pioneer within neuroscience, discovered that CNS axons failed to regenerate (21). Cajal demonstrated that axons from PNS, in contrast to those of the CNS, readily grew out and could re-innervate their targets following axotomy. He attributed the insufficient regenerative capabilities of CNS axons to their surroundings and hypothesized that the axons would grow if they were influenced by the same environment as peripheral nerves. To confirm this, he and his pupil Tello performed an experiment where they anastomosed a transected sciatic nerve graft to the optic nerve in a rabbit (22). They were able to demonstrate that some of the normally growth-resistant optic axons had penetrated and extended into the graft of the sciatic nerve. Cajal and Tello concluded that the environment surrounding the CNS axons at least were partially responsible for the poor regenerative capacity. However, the validity of these experiments were later questioned, as it was pointed out that the apparent extending CNS axons could represent autonomic fibers from blood vessels (23). It was not until 1981 that the field advanced, when the experiment by Cajal and Tello was repeated in a modified version by Aguayo and David (24). Using a mouse model, they grafted an autologous sciatic nerve between the medulla oblongata and lower parts of the spinal cord. After 22–30 weeks, horseradish peroxidase staining methods showed that axons had crossed from the CNS through the PNS graft penetrating the CNS tissue at the other end, where further growth stopped. This provided evidence that the mature CNS environment indeed inhibit axon regeneration, in contrast to the growth permissive PNS.

#### The role of myelin

The results sparked a search for biomolecules within the CNS responsible for preventing growth of injured axons. If one or more inhibiting factors could be identified, blocking their function should theoretically promote axonal growth. Focus was turned to myelinating cells, as it was shown that mature oligodendrocytes repelled axons and neurites in co-cultures *in vitro* (25). This observation led to the development of a monoclonal antibody against IN-1 on the oligodendrocyte surface, later known as Nogo-A. The blocking of Nogo-A allowed *in vitro* ingrowth of neurites into adult optic nerve explants (26). Another study demonstrated that the neutralization of Nogo-A promoted axon sprouting at the lesion site of the corticospinal tract transection in young rats (27).

**Figure 1:**
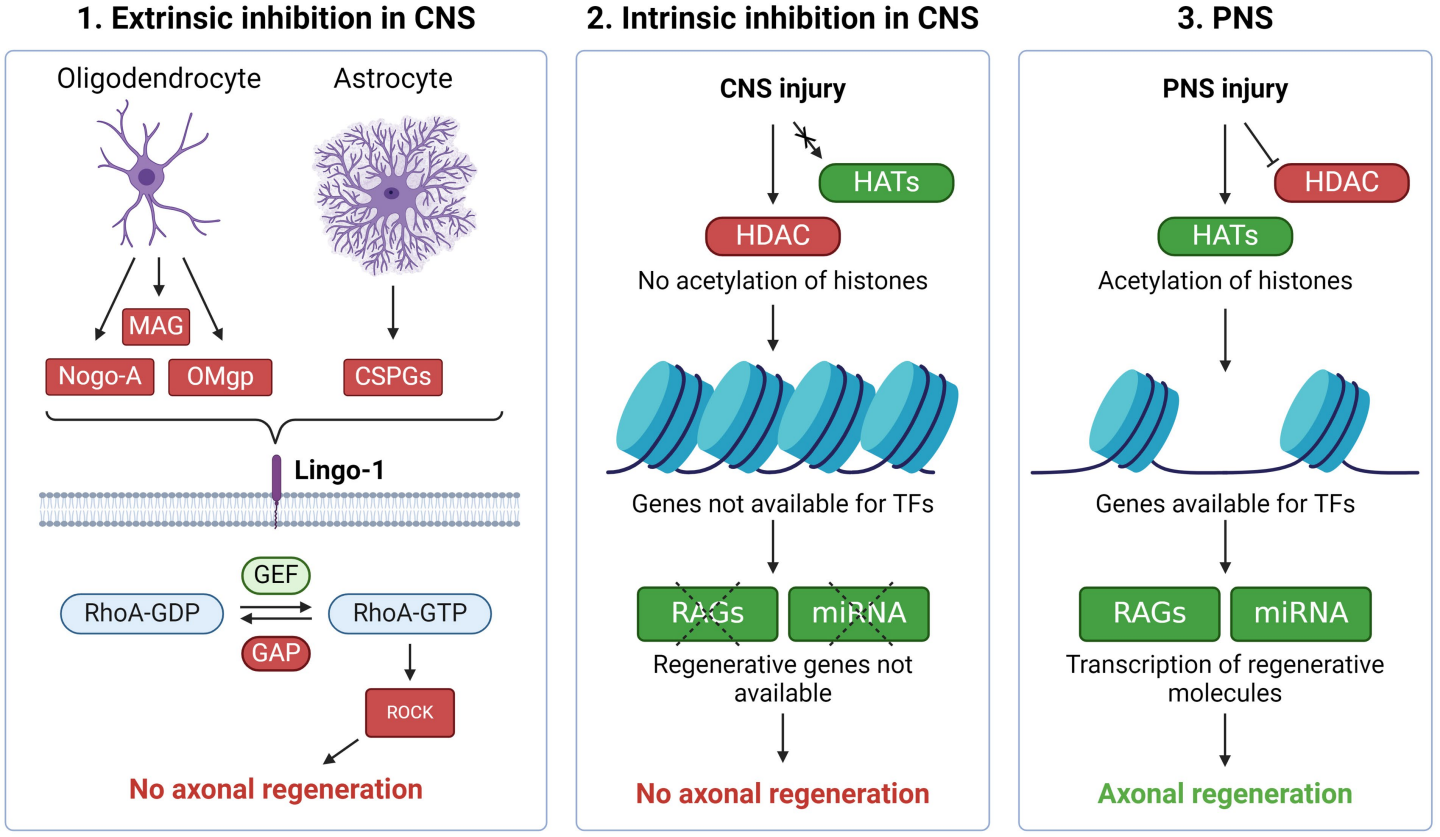
Regenerative inhibition in the CNS vs. PNS (1) Molecules produced by oligodendrocytes and astrocytes such as Nogo-A, MAG, OMgp and CSPG inhibit axonal regeneration through the Lingo-1 receptor. Activation of Lingo-1 facilitates phosphorylation of RhoA which in turn activates ROCK. The latter mediates axon growth inhibition through prevention of cytoskeleton- and growth cone formation. These processes are meant to stabilize the neuronal environment, resulting in the low regenerative ability present in the CNS. (2) In the CNS, epigenetic barriers also prevent axonal regeneration. After CNS injury, the acetylation needed to make regenerative associated genes (RAGs) available for transcription factors (TFs) do not occur, which in turn prevents axonal repair. (3) Upon injury in the PNS, histone deacetylases (HDACs) are rapidly exported out of the nucleus while histone acetyltransferases (HATs) start acetylation and thereby expose RAGs for TFs, resulting in axonal regeneration. Created with BioRender.com.

Subsequent studies identified other oligodendroglial proteins inhibiting axonal growth, including myelin-associated glycoprotein (MAG) (28) and oligodendrocyte myelin glycoprotein (OMgp) (29). These proteins were shown to impair axonal regeneration via activation of the Ras homolog member A (RhoA) and subsequently Rho-associated protein kinase (ROCK) (Figure 1) (30). ROCK mediates axon growth inhibition through phosphorylation of central molecules related to formation of the cytoskeleton (31). The importance of the myelin components in inhibiting axonal growth in the CNS was, however, later questioned when deletion of NOGO, MAG and OMgp failed to enhance axonal regrowth after SCI in knock-out mice (32). Although single deletion of each factor led to increased sprouting of uninjured axons, there was no associated behavioural improvement and no synergistic effect of deleting all three, suggesting additional contributing mechanisms responsible for the failing regeneration of the mature CNS.

#### Glial scar and inflammation

In concert with these findings, other inhibiting factors for axonal regeneration were identified, such as the glial scar (33). Following injuries to the CNS, reactive astrocytes form a gliotic scar at the lesion site. This process serves to isolate the inflammation and protect the surrounding tissue but comes at the cost of axonal regrowth. Proteoglycans, including chondroitin sulphate proteoglycans (CSPGs), are necessary for the formation of the scar. Similar to Nogo-A, MAG and OMgp, CSPGs prevent axonal growth via the same Rho/ROCK pathway (34). The inhibitive effect of CSPG was supported by studies demonstrating that the CSPG-digesting enzyme chondroitinase ABC leads to axon regrowth and functional improvement following CNS injury in animal models (35, 36). Although the glial scar forms a barrier to axonal regeneration, preventing its formation may also lead to increased lesion size and worse outcomes (37). Accordingly, it was shown that astrocytes can support axonal regeneration by the production of specific growth supportive types of CSPG (CSPG4 and CSPG5). These opposing findings highlight the heterogeneity of astrocytes and their impact on axonal regeneration.

### Intrinsic inhibition of regeneration

#### Implantation of embryonic neurons and the conditioning effect

Although it was clear that the environment of the CNS prevented axonal regrowth, there had to be additional unknown factors obstructing the process of regeneration. This was observed following the transplantation of fetal neurons into rodent models of SCI. After successful intralesional implantation, the cells survived up to 16 months in both neonatal and adult rats (38). Axons from the implanted fetal neurons crossed the entire length of the lesion and formed synapses with host neurons. The functionality of these connections were, however, not assessed, as the study was strictly histological and did not include neurological deficits as outcomes. Nevertheless, the findings suggested that immature neurons could overcome the inhibitory extrinsic environment, pointing at an additional intrinsic mechanism within mature neurons that, unlike fetal neurons, prevented axonal growth within the injured CNS. An intraneural switch seemed to exist that was turned off during development for the benefit of other functions than growth, such as synaptic activity. The intrinsic brakes of regeneration were illustrated by another sentinel experiment, which included an inflicted injury to the peripheral branch of dorsal root ganglion (DRG) neurons. DRG neurons are unique in the sense that they share the features of both PNS and CNS as the distal branch belongs to the PNS and the central branch to the CNS. While the peripheral axon of the DRG neuron under optimal circumstances regrow and re-innervate its target, the central branch fails to regenerate following axotomy. If, however, the distal branch is injured one week before the central branch, the central branch will also show signs of axonal regeneration. This phenomenon was named “the conditioning effect” and indicated that the intracellular modifications following peripheral nerve injury was sufficient to prime the CNS for axon regrowth (39, 40).

#### Regeneration-associated genes

Subsequent studies showed that axonal lesions in the PNS typically induce a broad and coordinated cascade of gene expression changes promoting regeneration, a cascade that is absent in injuries to the CNS (Figure 1) (41–43). These genes were named “regeneration-associated genes” (RAGs). Due to the broad apparatus necessary to initiate axon regrowth and elongation, RAGs code for several different types of proteins, including metabolic enzymes, cytoskeletal proteins and adhesion molecules. The specific function of each RAG has been determined using knock-out models or pharmacological inhibition in cell cultures as well as animal models (44, 45). These experiments have also demonstrated that not all genes expressed after PNS injury promote regeneration. Some may also inhibit regeneration, such as SOCS3. This protein is a suppressor of cytokine signalling and its overexpression leads to decreased axon growth (46). The fact that not all genes promote regeneration, illustrate the broad spectrum of gene networks that are activated in response to PNS axonal injury (47). An important question is whether the complete genetic response is necessary to sustain axonal regeneration within CNS neurons or not.

#### Master regulator transcription factors

Some transcription factors (TFs) are more important than others as they seem to regulate the activity of multiple RAGs. The deletion of these “master” TFs leads to a more substantial decrease in the regenerative response as compared to knockdown of individual RAGs (47). Importantly, the regenerative program orchestrated by the master TFs is only initiated following PNS injury, but not CNS injury (48). A study identified a total of 39 TFs as master regulators for around 400 signalling pathways in the regenerative response to peripheral sensory axonal injury (49). In later experiments, 23 additional master TFs were identified in rodent models of PNS injury (50). The master TFs are highly interconnected through multiple and parallel pathways, which contribute to the robustness of the response. Master TFs and their networks may thus represent potential targets for therapeutic modulation of axonal regeneration. Important master regulators include The Krüppel-like-factor (KLF) family, C-Jun, STAT3 and AKT. The use of multi-omics analyses has also brought new insights to uncover mediators of neural regeneration. Recently, a systems genomic approach showed that RE1-Silencing Transcription Factor (REST), also known as Neuron-Restrictive Silencer Factor (NRSF), is an important upstream suppressor of the pro-regenerative program in the CNS (51). In a mature mouse model of SCI and optic nerve crush, the deletion of REST led to upregulation of RAGs with improved axonal regeneration in both models. Another recent study applied multi-omics screening to identify the four TFs, ATF3, ATF3, C/EBPγ and SHOP/Ddit3 as central regulators of *in vivo* injury response following optic nerve crush in mice (52). Advances in sequencing technologies will likely continue to facilitate new discoveries to unveil the complex regulation of neural regeneration.

#### Epigenetic regulation

TFs initiate transcription of DNA by binding to their target sites. Epigenetic modifications of histones and DNA play an essential role ensuring chromatin accessibility for the TFs during axonal injury (53). Histone acetylation is the most studied mechanism during regeneration. Histones are acetylated by histone acetyltransferases (HATs) and deacetylated by histone deacetylases (HDACs). Generally, histone acetylation neutralizes the positive charge of lysine, which leads to increased accessibility, whereas deacetylation results in chromatin compaction and gene silencing. Both HATs and HDACs are involved in the neuroregenerative response. The HAT p300/CBP-associated factor (PCAF) is activated after PNS injury and modifies histones at key RAGs, increasing their accessibility (Figure 1) (54). In the absence of PCAF, there was no regeneration of CNS axons following a conditioning lesion. PCAF overexpression, on the other hand, promoted regeneration of sensory fibers in a mouse model of SCI.

With HDAC5, an opposite mechanism seems to promote regeneration. Following peripheral axotomy, the influx of calcium triggers exportation of HDAC5 out of the nucleus into the cytoplasm, which results in histone H3 acetylation and activation of a regenerative transcriptional program (55). This injury-induced nuclear export is essential for axon regeneration and fails to be activated in CNS injury.

Epigenetic modifications by DNA methylation also play a role in the regenerative response after axonal injury. DNA methylation is usually associated with suppressed gene expression, whereas hypomethylated regions become more accessible for transcription (56). Methylation occurs by DNA methyltransferases (DNMTs) and demethylation by ten-eleven translocation (TET) enzymes. PNS injury leads to upregulation of TET3 via calcium signalling and is necessary for RAG expression. After PTEN knock-out, axonal regeneration of mature retinal ganglion neurons, require activation of TET1, but not TET3. This may indicate that TET signalling is required for experimental induction of axonal regeneration in CNS, yet by implicating different DNA methylation pathways than the natural regeneration in the PNS (57).

An assessment of epigenetic patterns following axonal damage, showed distinct histone acetylation signatures correlating with gene expression associated with axonal regeneration (58). The correlation was only evident in the PNS, but not in the CNS. This points to an “epigenetic barrier” in the CNS neurons, which may, at least partly, explain its poor regenerative capacity. A similar barrier exists in PNS neurons, but is overcome following axonal injury due to epigenetic modifications (Figure 1). The barrier in the CNS appears during maturation of neurons as the axons reach their targets and form synapses (59, 60).

#### Post-transcriptional regulation

In addition to RAGs, master TFs and epigenetic modifications, post-transcriptional regulation of gene expression also plays an important role in the intrinsic regenerative program of the PNS. Micro RNAs (miRNAs) are a class of small (~22 nucleotides) noncoding RNAs that regulate post-transcriptional gene expression by preventing translation and/or promoting mRNA degradation (61). As each miRNA typically has several targets, multiple genes may be modulated simultaneously. Based on bioinformatic predictions, miRNAs may regulate >30% of all mammalian protein coding genes (62). These post-transcriptional modifications also appear to be a central player in axonal regeneration, as deletion of Dicer, an endoribonuclease essential for the assembly of miRNAs, leads to impaired axonal regeneration both in dissociated DRG cultures and in rodent models of PNS injury (63).

Specific miRNAs have also been shown to be highly involved in the regenerative response. In a rodent DRG model, mIR-21 was upregulated by 7-fold seven days after peripheral nerve branch axotomy (64). Overexpressed mIR-21 resulted in increased neurite outgrowth in dissociated adult rat DRG neurons, suggesting that mIR-21 is an axotomy-induced miRNA that promotes peripheral axon growth. In another study, mIR-26a mediated axon regeneration of sensory neurons in a sciatic injury model in mature mice via increased expression of GSK3β (65). In addition, miR-26a also supported neurite outgrowth in rat cortical neurons by suppression of PTEN (66). Furthermore, miR-133b promoted neurite outgrowth and functional recovery in a mouse SCI model by targeting the inhibitory RhoA (67). The different miRNAs involved in post-transcriptional regulation of RAGs and regeneration-associated cellular pathways may represent potential therapeutic targets in the promotion of neural regeneration.

#### Regeneration-associated cellular pathways

Several intracellular pathways are highly involved in the regenerative response to axonal injury. These include, but are not limited to, the pathways GSK3β/CRMP2 (68), JAK/STAT (69), DLK/JNK/cJUN (70), and cAMP/PKA/CREB (71). Describing each specific pathway would be beyond the scope of this review. Here, we briefly focus on the role of the PI3K/PTEN/mTOR and RhoA pathway as examples.

**Figure 2:**
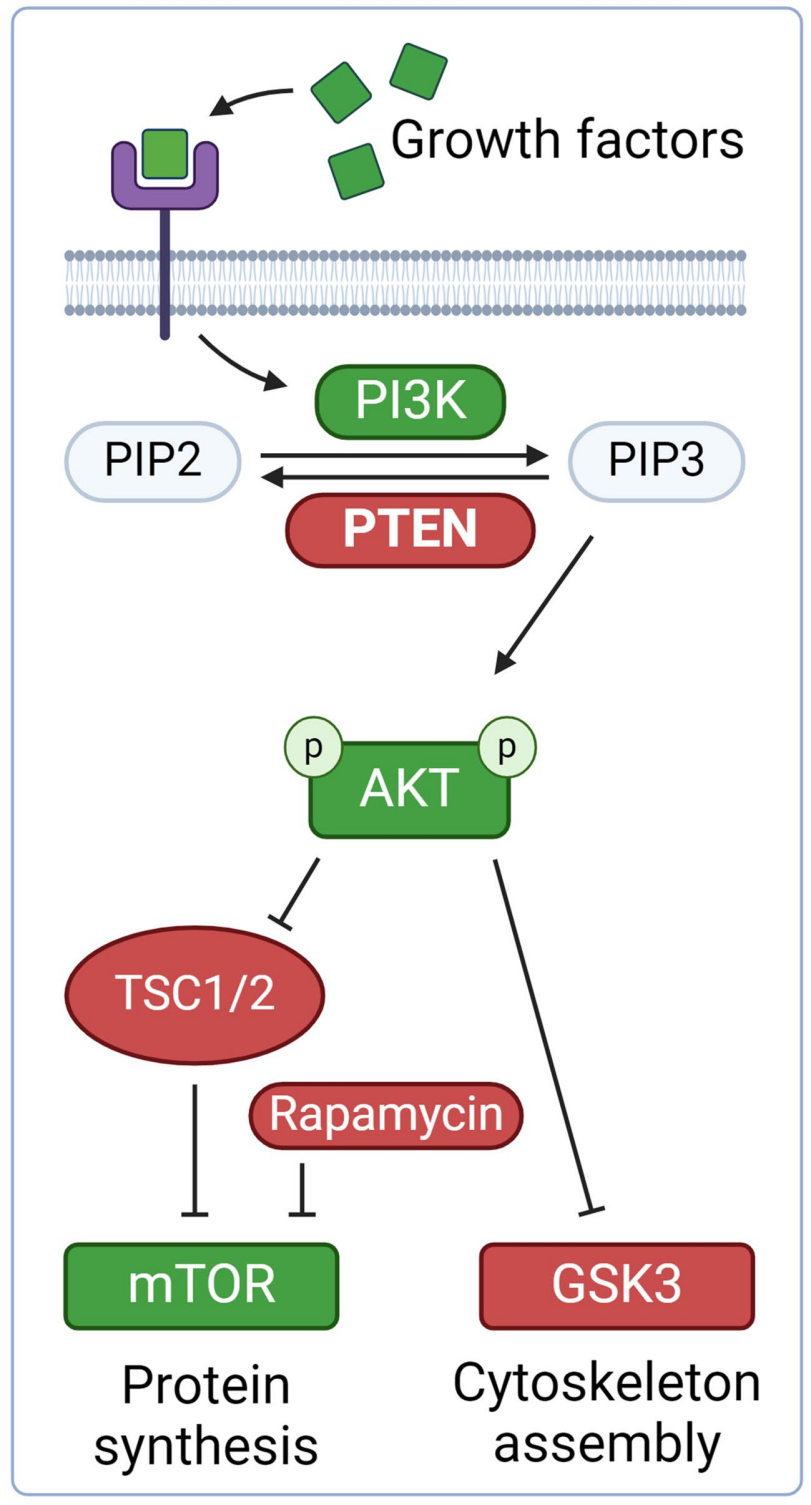
Neurotrophic growth factors, such as brain-derived neurotrophic factor (BDNF) and neurotrophin-3, bind to the tyrosine kinase receptor complex. This activates PI3K, which in turn converts PIP2 to PIP3, thereby phosphorylating AKT. AKT disinhibits mTOR via inhibition of the negative regulators TSC1 and TSC2. mTOR promote axonal regeneration through protein synthesis and cell growth. AKT also inhibit Glycogen Synthase Kinase 3 (GSK3), which promotes axonal growth. PTEN is an inhibitor of the PI3K/AKT/mTOR and thus prevents axonal regeneration. Created with BioRender.com.

The PI3K/PTEN/mTOR pathway is activated by binding of growth factors, such as brain-derived neurotrophic factor (BDNF) or neurotrophin-3 (Figure 2). This activates PI3K, which in turn converts PIP2 to PIP3, thereby phosphorylating AKT. AKT disinhibits mTOR via blocking of the negative regulators TSC1 and TSC2. mTOR regulates cell growth, protein synthesis and cell proliferation, and also promotes axon regeneration. PTEN is an inhibitor of the PI3K/AKT/mTOR pathway and leads to decreased levels of mTOR. In a pioneering experiment, deletion of PTEN promoted axon regeneration following optic nerve crush in a rat model (72). This effect was shown to be dependent of mTOR, as rapamycin, an inhibitor of mTOR, cancelled the effect of PTEN knock-out. The isolated activation of mTOR by TSC2 led, however, to less regeneration, suggesting that mTOR is not the only effector in the regenerative response of PTEN deletion. In a subsequent study, the same effect was shown in a rodent SCI model as PTEN deletion enabled injured corticospinal axons to regenerate past a spinal cord lesion (73). Ultrastructual analysis and staining with vGlut1, a presynaptic marker for excitatory synapses, suggested formation of synapses past the lesion. Regrowth of corticospinal tract lesions could also be induced by PTEN deletion one year after SCI in a mouse model (74). Another study showed restored locomotor abilities in both acute and chronic SCI in mice following PTEN knockout (75).

The small GTPase RhoA is a central binding element between extracellular factors inhibiting axon regeneration and the neuron-intrinsic response within the CNS. The myelin proteins MAG, NOGO, OMgp and the astroglial CSPG all activate RhoA, thereby preventing neural regrowth (34, 76). The consequences of RhoA activation are different in neurons and astrocytes (77). In neurons, RhoA restricts axonal growth by preventing microtubuli protrusion, whereas RhoA restricts injury-induced astrogliosis and CSPG production in astrocytes. Thus, blocking RhoA in neurons may promote regeneration, but cause opposite effects in astrocytes. This illustrates the complexity in tailoring regenerative therapies for axonal regeneration.

## Intra- and extracellular events required for neural regeneration

### Injury signalling

**Figure 3:**
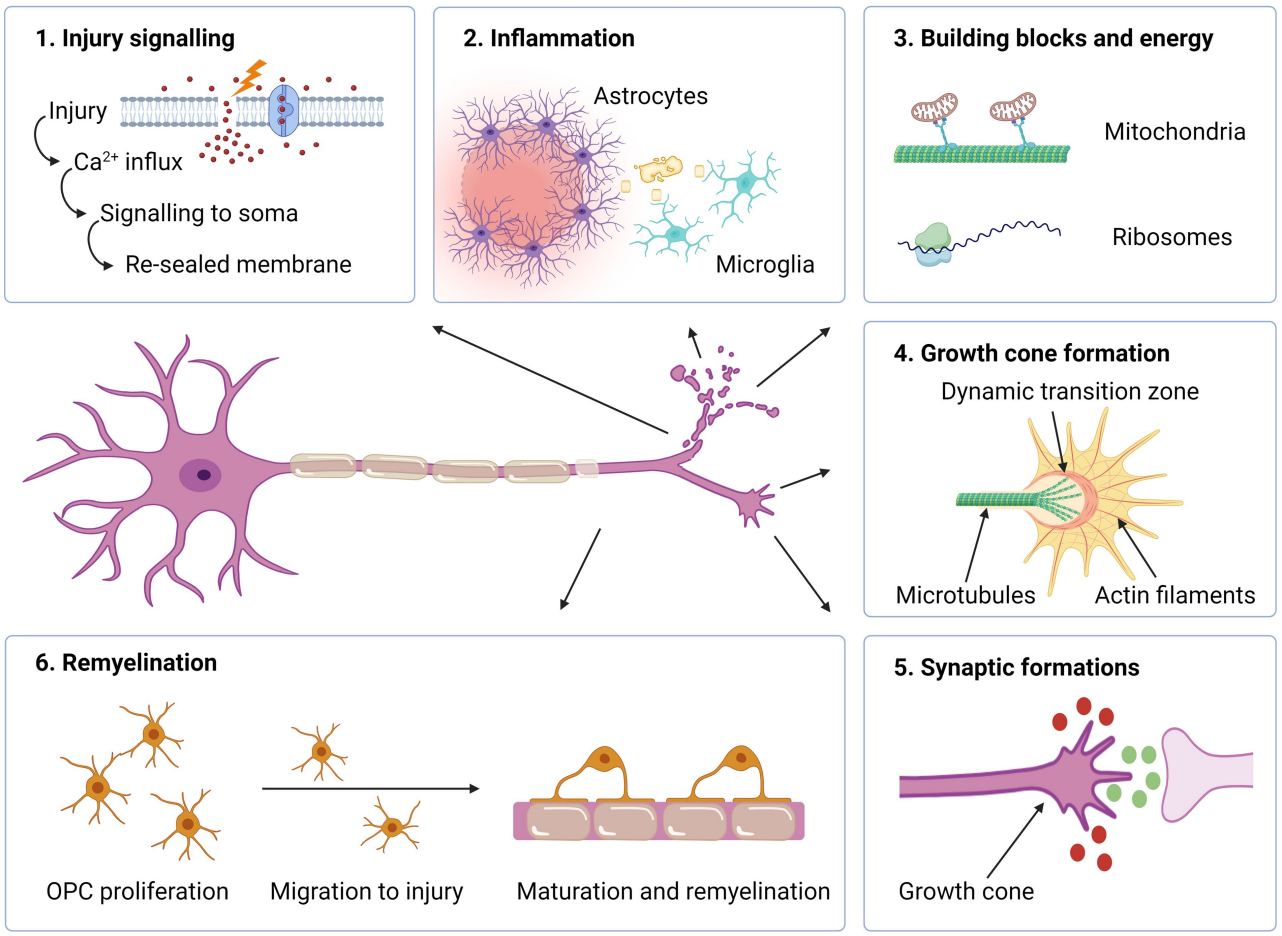
Intra- and extracellular events required for axonal regeneration. (1) Upon neuronal injury, influx of Ca2+ triggers signalling to the soma which in turn leads to an intracellular cascade with the increased levels of cAMP and- membrane resealing. (2) If the damage is profound, inflammation occurs where reactive astrocytes produce extracellular matrix molecules to form a barrier, thereby containing the damage. Microglia phagocytize myelin debris. (3) Mitochondria are transported towards axon tip for producing energy necessary for the regenerative process. Ribosomes located in the axon enable synthesis of proteins needed for the assembly of a growth cone. (4) In the axon terminal, a growth cone is formed, which enables axonal elongation. (5) Attracting (green) and repulsive (red) guidance molecules lead the way towards target synapses. (6) Oligodendrocyte progenitor cells (OPCs) proliferate, migrate and differentiate into mature oligodendrocytes, which remyelinate the nude, regenerated axon. Created with BioRender.com.

Signalling from the site of axonal injury to the soma and cell nucleus is essential for initiation of a regenerative response in neurons (Figure 3). In PNS, this is communicated to the cell body in two distinct phases. The first response is mediated by a fast retrograde calcium wave that propagates towards the soma at a speed of approximately 1 mm per minute (78). Calcium influx appears at the tip of the severed axon combined with the opening of voltage-dependent sodium channels and the inversion of Na+/Ca2+ pumps, alternatively through mechanophores in non-transecting trauma (79).

The calcium increase plays several important roles in the regenerative response, including membrane resealing, assembling a growth cone, and activation of signalling molecules and transcription factors (79, 80). Interestingly, the rise in intracellular calcium is so important for regrowth, that repair-competent neurons in a calcium-free environment fail to regenerate (81, 82). However, the rapid increase in calcium represents a two-sided blade, as excessive calcium levels may also initiate autodestructive mechanisms, which may lead to a breakdown of the cytoskeleton and apoptosis (83). Regulation and restoration of pre-injury intracellular calcium levels is thus an essential task in order to avoid cell death and promote a regenerative response. The response following axonal injury is also characterized by a slower retrograde transport of different signalling molecules, such as DLK (84). This pathway functions in a calcium-dependent manner and works as an important sensor for axonal injury (85) or cytoskeletal destabilization (86). Via downstream activation of the master TFs JUN and STAT3, the regenerative program with the expression of RAGs is initiated (70).

### Growth cone formation

The formation of a growth cone at the tip of the transected axon is an essential step in the process of regeneration. The transformation from a cut axon tip to growth cone involves major changes in the cytoskeleton, as well as the cell membrane and intracellular compartments. In PNS, the earliest regenerative sprouting occurs within 24 h, and usually within 3 h after injury (87, 88). In the growth cone, the actin-rich peripheral area is separated from the microtubule-rich central area by a dynamic transition zone where these two different domains interact (89). The peripheral part of the growth cone consist of sheet-like lamellipodia and finger-like filopodia, which sense and interact with the extracellular microenvironment. Mitochondria concentrate in the tip of the regenerating axon and produce the extra energy that is needed for axon growth (90). The growth cone is similar to the dynamic cone, which drives axon elongation during neural development (89).

The ability to form a growth cone at the end of the severed axon is a marked difference between CNS and PNS. Following axotomy of CNS axons, the bulbs typically fail to be organized into a growth cone. Instead, a retraction bulb is formed with disorganized microtubules and accumulating mitochondria and vesicles (87). Retraction bulbs have been identified more than four decades after spinal cord injury in humans (91). Some CNS axons may, however, under certain circumstances form growth-cone similar shapes. *In vivo* imaging of transgenic mice after single-cell axotomy, which produces minimal glial scarring, showed that almost 30% of the proximal stumps of sensory axons started to regrow during the first two days and that the distal segments resembled growth cones as they were spiked with numerous filopodia (92). Nevertheless, growth appeared at a much slower rate compared to PNS regeneration and the lesioned axons seemed to lack directional information as they grew laterally and made “U-turns,” forming large axon convolutes 1–2 weeks after injury.

Microtubules are important for the formation of a growth cone and axonal regeneration. If microtubules are stabilized by Taxol, a compound that promote the polymerization of tubulin and prevent depolarization of the microtubules, formation of growth cones are promoted. In a rat model of SCI, this also prevented scar formation and led to improved functional outcomes (93). Likewise, growth cones may be turned into retraction bulbs by pharmacological destabilization of microtubules using agents such as nocodazole, which reversibly interferes with the polymerization of microtubules (87).

### Building blocks and energy

The formation of a new growth cone and axonal elongation requires cellular components and large amounts of energy. As axonal injuries often occur at a distal location, several centimetres from the soma, the required material for regeneration must be either synthetized locally or transported to the site. The swift sprouting at the injured axon tip in the PNS suggests the first alternative. Indeed, mRNA and ribosomes have been located within distal PNS axons, thus enabling the synthesis of proteins needed for the assembly of a growth cone, such as actin and GAP-43 (94). The protein synthesis within the distal axons also produce signalling proteins, such as STAT3 and vimentin which are applied for retrograde signalling (95). Under normal circumstances, CNS axons have low levels of local translational capacity as compared to those in the PNS (81). This difference has been pointed out as an important factor restricting CNS regeneration. However, studies have shown that also CNS axons may have this ability (96). When a peripheral nerve segment was used as a growth-supportive substrate grafted into the transected spinal cord of adult rats, the regenerating CNS axons were shown to contain both growth related mRNAs as well as ribosome constituents. This suggests that CNS axons are able to generate the proteins necessary for growth cone construction and axonal elongation, if provided with an environment permissive for regeneration.

Following axotomy, growth cones increase the demand of energy, especially because axotomy depolarizes mitochondria and depletes ATP (97). In the CNS, around 20–30% of the mitochondria in neurons are motile and may be transported bidirectionally (98). The transport towards the axon tip occurs via a motor/adaptor complex on the mitochondrial surface containing the proteins Miro (RhoT1/2) and Milton (TRAK1/2), which link the mitochondria to the microtubule (99). The protein syntaphilin, on the other hand, immobilize the mitochondria by anchoring them to the axonal microtubules (100). Mitochondria transport occurs less in mature neurons than in developing neurons (99). Reduced mitochondrial motility may thus be an intrinsic mechanism inhibiting axonal regrowth in mature CNS neurons.

### Axonal elongation and synaptic formations

During development, the growth cone actively detects signals from the environment, and guides the elongation of the axon towards its target. In this complex and dynamic process, surface receptors at the finger-like filopodial projections of the growth cone interact with soluble or membranous guidance cues, such as netrins, semaphorins, Sonic hedgehoof and wnt (101). The signals are integrated within the growth cone, and cytoskeletal modifications made depending on whether the cues are attractive or repulsive. This induces movement of the growth cone in the right direction. A similar process occurs during axonal regeneration. Interestingly, however, the specific guidance cues can induce opposite effects in the mature CNS compared to the premature. This applies for Semaphorin3A, which is repulsive to embryonic DRG neurons, but promotes growth in mature DRG neurons (102). Other embryonic guidance molecules retain their inhibitory effect on the growth cone and some of these are expressed after injury to the mature CNS. Wnt proteins are good examples, as they repel the growth cone of descending axons during development (103). The decreasing concentration of Wnt proteins direct the axons from the brain in caudal direction towards the spinal cord. Following injury to the mature CNS, Wnt proteins are upregulated and inhibit axonal regeneration via the Ryk receptor. In a mature mouse model, blocking of Ryk with monoclonal antibodies enhanced the sprouting of the corticospinal tract branches around and beyond the injury site resulting in improved motor function (104). However, the positive effect was dependent on weekly task-specific training, as Ryk deficient mice without training did not achieve clinical improvement as compared to controls. This underlines the value of targeted rehabilitation on cerebral plasticity.

For the elongation of neural growth cones through areas of gliosis and fibrotic scar, it may be necessary not only to prevent inhibition by repulsive guidance cues, but also to attract the growth cones by the application of chemoattractive growth signals. A study involving a complete SCI model in mice and rats assessed the combination of (I) pre-injury boosting of neuron intrinsic growth capacity with the expression of osteopontin, insulin-like-growth factor 1 (IGF-1) and ciliary-derived neurotrophic factor (CTNF) via adeno-associated virus injections, (II) injection of depots containing fibroblast growth factor 2 (FGF2) and epidermal growth factor (EGF) for providing a growth supportive substrate within the injured core and (III) depots of glial cell line-derived neurotrophic factor (GDNF) for chemoattraction beyond the lesion (105). Individually, these treatments did not show any robust regenerative effects, but the combination of all three provided a strong propriospinal regrowth through the glial scar and a full spinal segment beyond the lesion center. The growing axons also formed synaptic-like contacts, which mediated a significant return of electrophysiological conduction across the lesion. However, the rodents did not improve over-ground locomotion, suggesting that new circuits formed after complete SCI need additional rehabilitation measures to provide a clinical benefit.

For achieving functional meaningful regeneration within the CNS, axonal elongation would have to be combined with the establishment of appropriate synaptic connections. Fortunately, it is not necessary to re-establish the exact synaptic connections as before the injury. Circuit reorganization and remodeling of spared neural networks may circumvent areas of pathology and replace the previous networks (106). This does not only apply for regenerating axons, but also for the sprouting unaffected axons. Training and rehabilitation may promote this process (107).

### Inflammation

Inflammation is involved in all pathological conditions affecting the CNS, including SCI, MS and IS. Within hours after CNS injury, activated microglia and astrocytes secrete inflammatory cytokines and chemokines, which increase permeability to the BBB and attract circulating leukocytes (108). The activated astrocytes isolate the site of injury by their hypertrophic cellular processes and production of extracellular matrix proteins. The inflammatory reaction may have both beneficial and detrimental effects on regeneration. Cytotoxic mediators from activated microglia and from peripheral immune cells typically accentuate tissue damage beyond the original lesion, thereby worsening clinical deficits. Concordantly, the depletion of peripheral macrophages was shown to improve outcomes with increased axonal regeneration and myelin preservation after acute SCI in a rat model, suggesting that macrophages are part of the inhibitory environment in CNS injuries (109). Subsequent studies have, however, shown that macrophages and microglia should not be considered uniform units. Microglia are typically divided into two phenotypes; the classically activated M1-phenotype, which contribute to a pro-inflammatory environment and M2, which is anti-inflammatory and promotes regeneration (110). The effect is mostly mediated via the secretion of cytokines, chemokines and growth factors. A similar classification has been applied for macrophages (111). Specifically, M2 microglia have been shown to release neurotrophic molecules and contribute to oligodendrocyte differentiation, remyelination and axonal regeneration *in vivo* (112, 113). This approach may, however, be oversimplified, as the M1 and M2 types likely represent the poles of a spectrum with various phenotypes in between (114).

Inflammatory cells also have an important function in the clearance of debris, which is necessary before any regeneration can occur (115). Prevention of macrophages from reaching the site of PNS injury in a rat model resulted in attenuation of myelin clearance and impairment of axonal outgrowth (116). Likewise, depletion of macrophages also led to impaired remyelination in both PNS (117) and CNS (118). Interestingly, macrophages and Schwann cells have been shown to clear myelin products more efficiently than microglia in the CNS (119, 120). As myelin proteins inhibit axonal regeneration and prevent differentiation of precursor cells into mature oligodendrocytes (121), the differences in myelin clearance capacity likely contribute to the improved regenerative capacity in the PNS as opposed to the CNS.

### Remyelination

In the CNS, axonal regeneration may not lead to sustained clinical benefits unless the axons are myelinated. Myelin, a specialized membrane synthesized by the oligodendrocytes in the CNS and Schwann cells in the PNS, is necessary for maintaining nerve conduction velocity and providing metabolic support to the underlying axon. Following injury to peripheral nerves, Schwann cells switch to a regenerative phenotype, forming tubular bands through which axons may regenerate (122). After repair has commenced, the Schwann cells change to a myelinating phenotype via changes in gene expression (123). Spontaneous remyelination can also occur within the CNS as long as the axon is not irreversibly damaged. The newly formed myelin is, however, thinner, shorter and more vulnerable than developmental myelin (124). The process of remyelination is important from a neuroprotective perspective, as remyelination is associated with prevention of axonal degeneration (125) and recovery of neurological deficits (126). In the demyelinating disease MS, there is a strong inverse correlation between the extent of remyelination and disability scores, demonstrating the neuroprotective effect of remyelination (127).

Demyelination models in animal studies have shown that CNS remyelination is typically performed by recruited oligodendrocyte precursor cells (OPCs), which account for 5–8% of the CNS cell population. Following a CNS injury, OPCs proliferate, migrate to the site of injury and differentiate into mature, myelinating oligodendrocytes (108). Pre-existing mature oligodendrocytes also participate in remyelination (128). In MS, remyelination typically occurs in the initial phase of the disease, but declines with increased disease duration and aging. OPCs are located in chronic MS lesions, but seem to be unable to differentiate into mature myelinating oligodendrocytes (13). It has been suggested that the blocking of OPC differentiation appears as a consequence of defective OPC recruitment, which results in OPCs reaching the axons too late and in numbers too few to initiate differentiation (129). The negative correlation between remyelination and ageing may be related to a decreased phagocytic ability of microglia, which is crucial for successful remyelination as myelin debris contains inhibitors of OPC differentiation (129). Impaired remyelination over time leads to axonal degeneration. It is thus possible that the declining ability of remyelination plays an important role in the transition from relapsing–remitting MS to progressive MS with a continued worsening of neurological deficits (13).

## Promotion of neural regeneration in spinal cord injury, multiple sclerosis, and ischemic stroke—strategies translated into clinical trials

### Stem cell-based treatments

Stem cell-based therapies offer a dynamic biological approach, with their ability to migrate to the site of injury and promote repair via multiple mechanisms. Especially neural stem cells (NSCs) and mesenchymal stem cells (MSCs) are relevant from a neuroregenerative perspective.

NSCs are self-renewing and multipotent cells with the ability to differentiate into astrocytes, oligodendrocytes and neurons (130). These stem cells may be obtained in an allogenic manner from fetal tissue, or autologously using cell reprogramming techniques. MSCs, on the other hand, are present in nearly all human tissues, including the bone marrow, where they serve to maintain the hematopoietic stem cell niches. MSCs do not have a unique cell marker and therefore represent a heterogenous population of stem cells (131). Nevertheless, they have the practical advantages of being relatively simple to obtain without major ethical issues and may thus be administrated autologously without the need of genetic modification or immunosuppression.

Studies have shown that both NSCs and MSCs, instead of (trans)differentiating into functional neurons or oligodendrocytes, mainly promote regeneration in non-canonical ways via their secretome and cell-to-cell interaction (132, 133). The regenerative and immunomodulating effects are mediated through secretion of cytokines and neurotrophic factors, such as nerve growth factor (NGF), BDNF, hepatocyte growth factor (HGF), and vascular endothelial growth factor (VEGF), in addition to exosomes containing mRNA and miRNA (134, 135). NCSs and MSCs have also been shown to be able to stimulate and promote the regenerative potential of the endogenous neural stem cells located in the subventricular zone, in the subgranular layer of the hippocampus and spinal canal (136, 137).

MSCs also possess immunomodulatory properties, with the ability to promote microglia to shift their polarization towards the regenerative M2 phenotype at the expense of the inflammatory type M1 (138). Applying these therapeutic mechanisms, NCSs and MSCs may “sense and react” in response to tissue damage in a more dynamic way than uniform pharmacological treatments that deliver a specific agent in a specific dose. In animal models, both NSCs and MSCs have been shown to be able to migrate and “home” towards chemokines released after CNS injury (139–141).

Clinical trials have been performed with the transplantation of both NSCs and MSCs in SCI, MS and stroke.

#### Spinal cord injury

In SCI, the goal of stem cell transplantation is to promote axon growth and remyelination, thereby restoring neural circuits and improving functional outcomes. Trials have been performed applying both NSCs and MSCs for these purposes. In a Swiss-Canadian trial, human fetal NSCs were administered to 12 patients with chronic thoracic SCI via intramedullary microinjections in an open neurosurgical procedure (142). All patients had a motoric-complete, sensoric-incomplete injury. After six years follow-up, the procedure was found to be safe. Although some segmental sensory improvements were noted in five patients, there were no significant motoric effects. Another open label study included 29 patients with subacute, cervical- or thoracic SCI in a dose-escalation study of intramedullary injected human fetal NSCs (143). A total of 11 patients had motoric and sensoric complete injury while 18 had motoric complete and sensoric incomplete injury. No safety concerns were related to cells or procedure and efficacy measures were not reported. In a subsequent trial applying the same cell product, a total of 12 patients with chronic SCI were included (144). In cohort one 15–40 million NSCs were administered intraoperatively to six patients in a dose-escalation design. In cohort two, additional six patients received 40 million NSCs, and efficacy was compared to four untreated controls. Although the treatment was shown to be feasible and safe, there were no significant clinical effects. The trial was prematurely terminated by the sponsor due to the findings in a pre-determined futility analysis.

The administration of MSCs have also been assessed in SCI, either via intrathecal or intraoperative injections (145). Similar to the trials assessing NSCs, most of these were open-label trials designed to show feasibility and safety without a control group. An exception was a randomized, controlled, double-blinded study, which investigated effects of intrathecally injected MSCs derived from umbilical cord in ten patients with chronic complete thoracic SCI in a cross-over design (146). At six months, the treatment was switched so that patients initially receiving MSCs now received placebo and vice versa. Apart from a significant increased pin-prick sensation in dermatomes below the site of injury, no improvements were noted in motor functions or neurophysiological parameters. Other types of cells have also been tested in patients with SCI, including a small phase I trials of Schwann cells (147) and case reports of olfactory ensheathing cells (148). No clear therapeutic benefits were reported.

#### Multiple sclerosis

Unlike in SCI and IS, lesions are multifocal in MS and undergo a chronic inflammatory and progressive neurodegenerative course. In theory, this may fit to the migratory capacity of NCSs and MSCs following intrathecal injection. Once the cells reach the lesions, their paracrine abilities can promote modulation of activated microglia and accelerate remyelination via stimulation of oligodendrocyte maturation. With intravenous administration, on the other hand, the cells are unable to reach the CNS as the majority get trapped in the lungs (149). Nevertheless, MSCs have been shown to provide systemic modulatory effects to the adaptive immune system, which hypothetically could have a beneficial effect in MS patients. Recently, the results of the largest study so far using MSCs in MS were reported (150). In this trial, 144 patients were randomized to receive either autologous bone marrow derived MSCs or placebo intravenously in a cross-over design. At 6 months, there were no difference in number of gadolinium-enhanced lesions, which was the primary outcome of the trial. Studies applying intrathecal injections have also been performed. Quite recently, the results of a “first in man,” phase I trial using fetal NSCs in 12 patients with progressive MS was published (151). This open-label study showed that one single intrathecal injection of NSCs was feasible and safe after 2 years follow up. All patients received immune suppression with tacrolimus during the study period to prevent unwanted immune responses and were randomized into four groups with increasing numbers of injected cells. Despite the small study population, exploratory analyses showed a dose-dependent decrease in brain atrophy on MRI and increased levels of neurotrophic factors in CSF, including GDNF, VEGF-C and stem cell factor (SCF), suggesting a beneficial effect. On the other side, there was a nearly significant worsening of EDSS during follow-up and 50% of the patients developed new brain lesions. The causality between these, both positive and negative, observations remain elusive as we do not know the fate of the cells once injected. In 2020, the results from the largest study so far using intrathecal administration of MSCs in MS were published (152). A total of 48 patients with active progressive MS were randomized to receive autologous BM-derived MSCs or placebo either intrathecally or intravenously in a cross-over design. There were no serious adverse events (SAEs) related to the cells or the procedure, and significantly fewer patients in both treatment groups (intrathecal and intravenous MSCs) experienced disease activity compared to those receiving placebo. Patients receiving MSCs also showed significant improvements in other tests, such as EDSS, 25-foot timed walking test, 9-hole peg test and OCT. Especially patients receiving intrathecal administrations had favourable outcomes. However, the study did not assess the underlying mechanism, i.e., how the MSC treatment led to clinical improvement. Consequently, it is not known whether the cells migrated to the lesions or how long they survived.

#### Ischemic stroke

Clinical trials have also been performed utilizing stem cell therapy aiming to improve outcomes following IS. In an open-label trial, 18 patients with chronic stroke received stereotactic implantation of modified allogeneic BM-derived MSCs (153). Although there were no SAEs related to the cells, six SAEs were caused by the invasive procedure. After one year of follow-up, modest improvements were noted in different clinical scales, such as the National Institutes of Health Stroke Scale (NIHSS) and the Fugl-Meyer total score. However, efficacy was difficult to interpret in the absence of a control group.

Allogeneic adult multipotent progenitor cells from BM have also been tested in IS. In a placebo-controlled, double-blinded phase II trial, 67 IS patients received these cells intravenously within 24–48 h after stroke onset (154). The rationale of the treatment was to provide peripheral immunomodulation, thus promoting neuroprotection by alleviation of the acute neuroinflammatory response. Although the treatment was safe and showed reduced serum levels of cytokines and regulatory T cells in patients receiving the cell product, no beneficial clinical effects were noted when compared to placebo.

Another trial assessed safety and efficacy of an immortalised human neural stem cell line in a dose escalation design with up to 20 million cells administered via stereotactic injection into the ipsilateral putamen (155). A total of 11 patients with chronic stroke and stable symptoms were included. After 2 years of follow-up, four SAEs were considered related to the procedure, but none directly to the cell therapy. Disability remained unchanged in seven patients, worsened in one and improved in three. A follow-up study with 23 patients applying the same principle of treatment showed similar results and reported improvements in upper limb function in patients with residual upper limb movements at baseline, thus identifying a possible target population for a larger study (156). A phase IIb trial was initiated, but reported terminated in 2021 (NCT03629275).

Recently, another two randomized, controlled trials including 39 and 17 stroke patients failed to show improved clinical outcomes after receiving autologous MSCs, as compared to placebo (157) or those receiving standard care (158).

### Other therapeutic agents supporting neuroregeneration

The complexity of the mechanisms preventing regeneration following CNS injuries, enables several potential targets of therapeutic impact. In general, pharmaceutical agents may either provide neural regeneration by blocking inhibition or by enhancing regeneration-promoting factors. Therapeutic examples from animal studies that prevent inhibition include enzymes neutralizing CSPG molecules, Rho inhibitors and NOGO antibodies, whereas the application of neural growth factors and miRNA may directly stimulate neural regeneration. Several promising agents from pre-clinical experiments have not reached the stage of clinical trials due to different reasons, including the safety aspect. Blocking PTEN provide a good example, as deletion or inhibition of PTEN results in robust axonal regeneration, but also carry an increased risk for oncogenesis. These aspects may delay or prevent translation into clinical trials. Some therapeutic agents have, however, been tested in human patients.

#### Spinal cord injury

An example of targeting inhibitory mechanisms preventing neural regeneration is VX-210. This is a derivate of the bacterial enzyme C3 transferase, which inhibits Rho activity through covalent modification. As previously described, Rho activation stalls neural regrowth by promoting the collapse of the axonal growth cone. In mouse models of SCI, VX-210 (in the study referred to as “BA-210”) was applied to the dura mater and diffused into the spinal cord (159). Rho was deactivated in a dose-dependent manner and locomotion was improved when VX-210 was administered at the time of injury or 24 h post-injury. A similar approach was tested in a phase I/II dose-escalation trial, which included 48 patients with complete SCI at the cervical or thoracic level (160). Here, VX-210 was topically applied in a fibrin sealant to the dura mater following decompressive surgery ≤7 days after injury. Results confirmed safety and suggested improved motor strength as compared to historical controls in patients with cervical, but not thoracic SCI. A larger trial commenced, but was pre-maturely terminated due to fulfilment of predefined futility criteria after the inclusion of 70 patients (161). Heterogeneity of the patient population and suboptimal administration mode were among the highlighted factors potentially explaining the negative results. Nevertheless, this was the first randomized trial to assess an agent designated to block an inhibitory factor of neural regeneration.

In SCI, another clinical trial was performed using ATI355, a recombinant human antibody directed towards the human Nogo-A protein (162). This notion was based on the observation that intrathecally delivered Nogo-A antibodies promoted axonal growth and functional recovery in rodent and primate models of SCI (163, 164). A total of 52 patients with acute and subacute complete SCI received intrathecally administrated ATI355, either via continuous infusion or repeated spinal injections over four weeks in different doses. The Nogo-A antibody was generally well tolerated, although one case of bacterial meningitis was reported as a SAE related to administration method. The concentration of the ATI355 in CSF was mostly around 0.1 μg/mL, which should have been sufficient for therapeutic effect based on pre-clinical data. Efficacy parameters did not show any clinical improvements as compared to retrospective longitudinal data. The study was, however, not primarily designed to assess clinical efficacy.

In SCI, the loss of tissue and cystic cavity represent a substantial hurdle for regeneration and functional recovery. Polymer-based biomaterial scaffolds have been associated with prevention of cystic cavitation, less glial scarring and axonal sprouting through the scaffold in rodent and non-human primate SCI models (165, 166). The implantation of such a device within the cavity of SCI has also been assessed in a clinical study. An open-label trial treated 19 thoracal SCI patients requiring open spine surgery with a bioresorbable polymer device (Neuro-Spinal Scaffold) within 96 h postinjury (167). Patients were followed for two years, and the procedure was safe and well tolerated. Although the trial did not have a control group, comparison to historical controls indicated clinical benefit with 32% of patients converting to motor incomplete injury as opposed to ≤17% in historical data. Nevertheless, a placebo-controlled trial is required to further evaluate efficacy.

#### Multiple sclerosis

In MS, clinical trials have aimed for remyelination, thus promoting neural regeneration indirectly. One therapeutic option has been to target the Lingo-1 receptor (Figure 1). This glycoprotein is selectively expressed on CNS neurons and oligodendrocytes and inhibits oligodendrocyte differentiation, myelination and axonal regeneration (168). Opicinumab, a human monoclonal antibody against Lingo-1, has been shown to promote remyelination in rodent demyelination models (169). A randomized, phase II trial of 82 patients with a first episode of optic neuritis suggested improved optic nerve conduction of opicinumab, although not statistically significant. The findings led to a larger phase II trial, which included >400 patients with MS (170). Patients were treated with intravenously administrated opicinumab or placebo every 4 week over 72 weeks in a dose-escalation design. Results failed to show a dose linear improvement of disability, which was the primary endpoint. The highest dose of 100 mg/kg, being applied in the previous trial of optic neuritis, did not prove efficient as compared to placebo. However, the doses 10 mg/kg and 30 mg/kg showed some effect in favour of opicinumab. The authors discussed the possibility that a too high dose of opicinumab could have differentiated the oligodendrocytes too early, making them unable to migrate into the demyelinated lesions.

Retinoic acid receptor RXR-gamma agonists have also been shown to promote remyelination in models of demyelination *in vitro* and *in vivo* (171). The retinoic X receptor is a positive regulator of OPC differentiation and may thus mitigate the impaired maturation of OPCs in chronic MS lesions (172). Bexarotene, a retinoid and an antineoplastic agent used to treat cutaneous T cell lymphoma, is an agonist to the gamma retinoid X receptors. A randomized placebo-controlled phase II trial was performed to assess the remyelinating potential of bexarotene in MS (173). Results after inclusion of 52 patients showed no differences in remyelination as assessed by mean lesional magnetisation transfer ratio on MRI at 6 months. The treatment was not well tolerated as there were significantly more adverse events in the treatment group than in the placebo group.

Finally, clemastine has also been tested for remyelination in a clinical setting. Clemastine is a first generation antihistamine available over the counter in most countries and has been shown to induce OPC differentiation and myelination due to off-target antimuscarine effects in pre-clinical demyelination models (174, 175). In a phase II trial, 50 patients with relapsing remitting MS and chronic demyelinating optic neuropathy were randomized to clemastine fumarate for 90 days and placebo for 60 days in a cross-over design (176). Results showed a significant decrease in latency delay in visual evoked potentials, suggesting a remyelinating effect. There were no differences in secondary endpoints, such as MRI parameters or clinical measures, including EDSS, T25W and 6MWT. Fatigue was a common adverse event, possibly related to the antimuscarin blockade. So far, no confirmatory phase III trials have been performed. Recently, the clemastine arm in an ongoing interventional trial was terminated because three patients with progressive MS experienced increased disability during treatment (177). CSF proteomic profiling showed that clemastine may have caused innate inflammation via the purinergic P2RX7 receptor in microglia and oligodendrocytes. This discrepancy highlights important differences in treatment effects between progressive and relapsing-remitting MS.

#### Ischemic stroke

In IS, treatment with cerebrolysin has been translated into the stage of clinical trials. Cerebrolysin is a mixture of enzymatically treated peptides derived from porcine brain tissue with proposed neurotrophic properties (178). The therapeutic effect has been suggested to occur through enhanced neurogenesis involving the sonic hedgehog pathway leading to improved outcomes in rodent stroke models (179). Other mechanisms include reduced levels of free radicals dampening excitotoxicity. In an exploratory, placebo-controlled clinical trial including 208 stroke patients, cerebrolysin was intravenously administrated within 24–72 h after stroke onset and continued for 21 days (180). The cerebrolysin group showed improved upper limb function at day 90 with good tolerability. A follow-up study did, however, not show any significant clinical effects (181). A meta-analysis including 1,417 trial participants, could also not demonstrate clinical benefits of cerebrolysin in the treatment of acute IS (182).

Granulocyte-colony stimulating factor (G-CSF) is another agent that may promote neurogenesis after stroke, thereby reducing stroke volume and inducing functional neurological improvement (183). G-CSF increases the release of myeloid cells and hematopoetic stem cells from the bone marrow and is frequently applied following chemotherapy-induced neutropenia and stem cell transplantation. The potential neuroregenerative effect in stroke is thought to occur via G-CSF receptors on neural progenitor cells, stimulating their differentiation into neurons (184). Although an initial study showed safety and a promising dose-dependent effect in acute IS (185), a subsequent phase IIb placebo-controlled trial including 328 patients failed to show efficacy both to clinical outcomes and imaging biomarkers (186). In this trial, IS patients received G-CSF intravenously over 72 h within 9 h after stroke onset. A following meta-analysis did not show improved outcomes in stroke patients receiving G-CSF, but a trend towards more frequent SAEs (187).

## Conclusion and perspectives

The field of research within neuroregeneration has come a long way from the Egyptian observations documented on papyrus some 4,000 years ago. We now know that both intrinsic as well as extrinsic mechanisms prevent axonal regrowth after CNS injury. The path of regeneration seems to have been closed during evolution for the protection of the complex mammalian CNS. Instead, a strategy of plasticity and reorganization of synapses from surviving neurons has evolved. In most cases, however, this strategy is only modestly successful in promoting functional improvement following CNS injury. Nevertheless, there must be notable benefits restricting regeneration and choosing plasticity instead, given that this has been the path of mammal evolution. With this backdrop, it should not come as a surprise that it is hard to promote regeneration within the CNS, and the fact that there is no such thing as a neuroregenerative treatment. Both cellular and pharmacological agents have started to translate into clinical trials, but overall results so far have been disappointing with no studies showing consistent efficacy. The negative results contradict the promising findings in animal studies applying different models of SCI, MS and IS. This discrepancy may be related to several factors. Firstly, neural regeneration is complex. Still, we do not know the exact molecular mechanisms of the failing regenerative process in neurons and glial cells within the CNS. Without this knowledge, how can we expect to develop an effective treatment? Secondly, there are many hurdles in the translatory process going from promising pre-clinical results into clinical efficacy. Poor methodology, low statistical power and publication-bias may contribute to the failure of translating potentially pro-regenerative treatments into human patients. In addition, SCI, MS and IS typically affect patients with a mature CNS. Animal models often apply young or adolescent individuals with more regenerative potential. Human “real-life” CNS conditions are also far more heterogeneous than induced disease models in animals and outcomes may be trickier to assess. This may lead to a need for higher power to show effect and the possibility of type II errors with negative results despite a real biological effect. Thirdly, and perhaps most importantly, no single intervention may be capable of overcoming the robust intrinsic and extrinsic barriers that prevents regeneration in the human CNS. A combination is probably necessary to circumvent the inhibitory mechanisms and simultaneously stimulate appropriate growth, as shown in the mentioned experiments of Anderson et al. with axons propelling through rodent models of complete SCI (105). But as the results demonstrated, this is not enough to provide actual functional benefits. Myelination is also needed to allow efficient flow of action potentials, in addition to training and rehabilitation for circuit remodelling and augmentation. At the same time, aberrant axonal growth must be avoided to prevent neuropathic pain syndromes and epileptic seizures. These combinatory issues, providing efficacy with clinical benefits and acceptable safety profile, represent a formidable task for future neuroregenerative trials. New potential therapies include gene editing with CRISPR-based technology to modulate the genetic machinery following neural injury (188), the use of artificial or stem cell-derived exosomes to deliver miRNA for immunemodulation and promotion of regeneration (189) as well as electric stimulation for remyelination (190). These upcoming approaches have, however, not yet been properly assessed in clinical trials of CNS injury. In the meantime, the need for regeneration may be outpaced by todays accelerating digital innovation. Recently, a Swiss group showed that the implantation of a brain-spine interface with epidural electrical stimulation resulted in a regained ability to stand, walk, climb stairs and even traverse complex terrains in a patient with incomplete, cervical SCI (191). Brain-computer interfaces may allow patients to control almost any electronic device by cognition (192). This technology is most likely in its infancy and has an enormous potential to improve everyday lives of many. The future will show whether the quest for neural regeneration within the human CNS will be solved or made redundant by digital innovation.

## Author contributions

CK: Writing – review & editing, Writing – original draft, Visualization, Validation, Software, Resources, Conceptualization. TK: Writing – review & editing, Writing – original draft, Visualization, Software. SG: Conceptualization, Writing – review & editing, Writing – original draft. LB: Conceptualization, Writing – review & editing, Writing – original draft.
